# Geographic Proximity to Parents, Intergenerational Support Exchange, and Migration Within Germany

**DOI:** 10.1007/s10680-020-09558-w

**Published:** 2020-03-17

**Authors:** Bettina Hünteler, Clara H. Mulder

**Affiliations:** 1 Institute of Sociology and Social Psychology, University of Cologne, Albertus-Magnus-Platz, 50923 Cologne, Germany; 2 Population Research Centre, Faculty of Spatial Sciences, University of Groningen, Landleven 1, 9747 AD Groningen, The Netherlands

**Keywords:** Internal migration, Family ties, Intergenerational support, Spatial proximity, Parent–child relationship

## Abstract

Previous research on internal migration has emphasised the importance of local ties to family members outside the household, and to parents in particular. Family members who live close to an individual’s place of residence represent a form of local social capital that could make migrating costlier, and therefore less likely. This idea has been empirically supported. Yet, how family ties bind remains largely unexplained. We assume that intergenerational support is a manifestation of local social capital, and that spatial proximity is needed for support to be exchanged. Thus, we used mediation analysis that includes explicit measures of support exchanges between parents and their adult–children born in 1971–1973, 1981–1983, and 1991–1993 to explain the binding effect of living close to parents. Logistic regression models of migrating a distance of more than 40 km were conducted using eight waves of the German *pairfam* data. Living close to one’s parents was indeed found to be negatively associated with the likelihood of migration, and part of this association could be explained through intergenerational support: the more the instrumental support an adult child exchanged with her/his parent, the less likely she/he was to migrate. Receiving emotional support from the parents was associated with an increase in migration propensity. Neither giving emotional help nor receiving help with childcare functioned as mediators. It thus appears that adult children are particularly likely to value the proximity of their parents when they are exchanging instrumental support, but that the emotional bond between adult children and their parents can often be maintained over longer distances.

## Introduction

Internal migration—that is, a long-distance move within a country—is often seen as a way for people to increase their spatial flexibility and widen their options for pursuing paid work and higher education. At the same time, migration is costly for the mover because she/he has to leave behind social and economic capital at the location of departure. A large body of research has focused on the economic determinants of internal migration. Social factors have also been found to play a part in internal migration. As a rapidly expanding body of the literature has shown, living in close spatial proximity to family members appears to greatly decrease the probability of migrating (Kan 2007; Michielin et al. 2008; Mulder and Malmberg 2011, 2014; Mulder and Wagner 2012; Clark et al. 2017; Ermisch and Mulder 2018). While the importance of family ties in migration decisions seems to be broadly acknowledged, the processes that underlie this relationship are less clear. The decrease in the likelihood that people will migrate if their parents live nearby seems to be partly explained by actual face-to-face contact between parents and their adult–children (Ermisch and Mulder 2018). However, the question of why this contact matters has yet to be answered.

In this article, we make a further attempt to disentangle the association between local family ties and the probability of migrating longer distances within a country. It may be assumed that the exchange of intergenerational support contributes to an individual’s social capital, which is understood as resources provided through social networks (see Kan 2007). We therefore investigate how several dimensions of support exchanges, particularly the instrumental and emotional dimensions and childcare provided by the grandparents, might mediate, and thus (partly) explain, the negative association between spatial proximity to the parents and the probability of migrating.

We perform our analyses for Germany. Compared with other European countries, Germany has moderate internal migration rates (Bell et al. 2018) and slightly longer distances between parents and adult children (Isengard 2013). It has an intermediate position on the North–South gradient with regard to frequent grandparental childcare provision by the grandmother (Attias-Donfut, Ogg, and Wolff 2005) as well as help and care given by adult children to their parents (Brandt et al. 2009). Family policies in unified Germany have been assigned to the category of gendered familialism (e.g. Leitner 2003; Krapf 2014), with no strong tradition of formal childcare in West Germany but traces of a history of abundant childcare in the East (Goerres and Tepe 2012; Schober and Spiess 2015).

The following research questions are addressed: To what extent is an adult child’s likelihood of migrating within Germany associated with living close to her/his parents? To what extent is this association mediated by the frequency of exchanges of instrumental and emotional support, as well as downward flows of caregiving, between parents and their adult children when they live close to each other? We conduct a mediation analysis through logistic regression models using Waves 2–9 of the German partnership and family panel *pairfam*.

## Theoretical and Research Background

It is generally assumed that individuals migrate only if the expected subjective benefits of the migration exceed the costs (DaVanzo 1981). Location-specific capital of an economic or a social nature, also denoted as local ties (e.g. Mulder and Wagner 2012; Mulder and Malmberg 2014), is an asset that an individual cannot or would find difficult to take to another location (DaVanzo 1981). As giving up location-specific capital is associated with considerable costs (DaVanzo 1981), people are less likely to migrate if they have such capital (David et al. 2010; Mulder and Wagner 2012).

Previous research has suggested that family members, and parents in particular, are among the primary sources of social capital (Ermisch and Mulder 2018) because they are major providers of support for their adult children (Bengtson 2001). Therefore, living close to one’s parents is an important deterrent to migration (Michielin et al. 2008; Mulder and Malmberg 2011, 2014; Mulder and Wagner 2012; Ermisch and Mulder 2018). It has, for example, been shown that individuals and couples in Sweden and the UK were significantly less likely to move long distances of 40 or 50 km if they were living within 2 km of their parents (Mulder and Malmberg 2014), or within a travel time of 1 h (Ermisch and Mulder 2018). Research conducted in the Dutch context has found that the location of the parents seemed to become especially salient when the adult children’s needs increased, such as upon divorce (Michielin et al. 2008). When co-residing couples were separating, having at least one parent living in the municipality of the formerly shared home was shown to be negatively associated with the likelihood of moving (Mulder and Wagner 2012). Partners of formerly co-residing married couples or co-residing couples with children in Sweden have been shown to be deterred from moving out of their shared home and from moving longer distances when they had family ties to their own mother, father, or siblings (Mulder and Malmberg 2011). In a first step, we want to replicate these empirical findings of a negative association between living in close geographic proximity to one’s parents and the likelihood of migration for the societal context of Germany. Following the theory of location-specific capital (DaVanzo 1981), we hypothesise that *individuals living in close geographic proximity to their parents are less likely to migrate than individuals who do not live close to their parents* (**H1**).

Next, we seek to explain (part of) this association. Ermisch and Mulder (2018) directly measured adult children’s ties to their parents by accounting for the frequency of their face-to-face contact. They found that the smaller likelihood of moving more than 40 km for children who lived close to their parents was indeed partially explained by frequent contact. We extend this finding by paying closer attention to the multi-dimensionality of intergenerational relationships (Bengtson and Roberts 1991) and by focusing on the exchange of support between parents and their children. Giving and receiving intergenerational support is known to require spatial proximity and face-to-face contact in most cases (Knijn and Liefbroer 2006; Hank and Buber 2009; Mulder and Van der Meer 2009; Zhang et al. 2013; Clark et al. 2017). Therefore, for individuals who live close to their parents, frequent exchange of support should make migration unlikely because migration would increase the distance to the parents.

Different kinds of intergenerational support exchanges might deter adult children from migrating to a greater or lesser extent. Providing and receiving instrumental support, such as helping a relative with household tasks (e.g. Van Gaalen and Dykstra 2006), are almost impossible without being physically present (Knijn and Liefbroer 2006; Isengard 2013). Participating regularly in these kinds of intergenerational support exchanges should make it more difficult for an adult child to migrate if they live close to their parents. We hypothesise that *the frequency of instrumental support exchanges between parents and their adult children partially mediates the relationship between spatial proximity to the parents and the likelihood of migrating. The more frequently instrumental support is exchanged, the lower the likelihood of migrating for individuals living close to their parents* (**H2**).

One particular component of such instrumental support exchange is intergenerational caregiving. In fact, caregiving from the parents of adult children for their grandchildren seems to be one of the most frequent kinds of intergenerational support flows (Knijn and Liefbroer 2006). Like instrumental support in general, intergenerational caregiving requires physical presence. Therefore, living spatially close to one’s parents is also almost a precondition for the provision of care (Pink 2018, for instance). Particularly when the need for functional or instrumental support increases, parents and their children are likely to move closer to each other (e.g. Rogerson et al. 1997; Pettersson and Malmberg 2009; Smits 2010). The need for care has also been shown to be a particularly strong deterrent to moving away from family members (Michielin et al. 2008; Hank and Buber 2009). While Michielin et al. (2008) proxied the potential need for intergenerational support through life events, such as childbirth or the ageing of a parent, we aim to directly assess the extent to which these needs are fulfilled and to include these measures in our analysis. We hypothesise that *the frequency of grandparental childcare partially mediates the relationship between adult children’s spatial proximity to their parents and their likelihood of migrating. Individuals whose parents are providing care for their children are less likely to migrate than individuals who are not receiving childcare support from their parents if they live close to them* (**H3**). Due to data restrictions, we are not able to investigate separately the role in migration decisions of care provided by the adult child to the parent, but we include this form of care in our measurement of instrumental support.

In contrast with instrumental support exchanges, emotional support, such as involvement in the personal life of the parent or the child (e.g. Van Gaalen and Dykstra 2006), might to some degree be provided through the use of information and communication technologies, such as the Internet and smartphones (Sharaievska 2017). Face-to-face contact might therefore be less of a precondition for the exchange of emotional support. We thus expect that *the frequency of emotional support exchanges between parents and their adult children partially mediates the relationship between the adult children’s spatial proximity to their parents and their likelihood of migrating, but less strongly than instrumental support does. The more frequently emotional support is exchanged, the lower the likelihood of migrating for individuals living close to their parents* (**H4**).

The direction of the relationship between geographic proximity to the parent and the likelihood of migrating might partly also be the opposite of the direction we hypothesised. For example, those living close to parents might have moved close to them to provide support (see Michielin et al. 2008, who examined the location of parents as a factor that can both discourage and encourage migration). Moreover, unobserved characteristics of the respondents, such as the value they attach to close family relationships, might have influenced the proximity to the parents, the mediators, and the likelihood of migrating. This means that, strictly speaking, the results cannot be interpreted causally. In the discussion section of this paper, we address this issue in more detail.

## Data and Methods

### Data and Sample

We use longitudinal data drawn from Waves 2–9 of the German family panel *pairfam* (Brüderl et al. 2019a). Pairfam is a multi-actor panel study focusing on partnerships and family relationships that has been conducted annually from 2008 to 2009 onwards. The survey data cover not only the primary respondent, but also target the respondent’s partner(s), up to three of her/his (step-)parents, and her/his children as respondents (Huinink et al. 2011). For our analysis, we only used data from the primary respondents. Pairfam contains extensive information on intergenerational relationships and support exchanges, as well as continuously updated data on changes of residence and information on distances between the places of residence of the respondent and of her/his parents (Brüderl et al. 2019b). Following Ermisch and Mulder (2018), we analyse the association between intergenerational relationship characteristics and the probability of migrating between two subsequent years. We cannot analyse migration behaviour in relation to intergenerational relationship characteristics for every wave because questions on support exchanges are included only in every second wave from Wave 2 onwards (Thönnissen et al. 2019). Therefore, the analyses are based on data from wave pairs 2 and 3, 4 and 5, 6 and 7, and 8 and 9. Migration behaviour—the dependent variable—is measured between every even wave *t* and every uneven wave *t* + 1. The independent variables are assessed at every even wave *t*. The baseline sample contains a total of *N* = 23,449 person-wave observations of *n* = 8028 different individuals who participated in any *t* and did not attrite afterwards. In pairfam, respondents can skip participation in one wave and then re-participate in the next wave without being eliminated from the panel (Suckow et al. 2010).

Pairfam is a cohort study that includes respondents from three birth cohorts: 1991–1993, 1981–1983, and 1971–1973. We excluded person waves in which individuals were co-residing with either of their parents (*n* = 7993 dropped). Naturally, most of these belonged to the youngest cohort (85.49%). The situation with regard to support exchange is very different for those living with their parents compared with those living away. Those living with their parents receive parental support almost by definition, but the support has a distinct character (housing provision, daily interaction). Furthermore, migrating out of the parental home is a form of leaving the parental home—a distinct type of move related to the transition to adulthood. Only local ties to biological parents were considered (*n* = 73 dropped). Cases in which individuals migrated out of Germany (*n* = 19) or back from a foreign country (*n* = 18) or the respondent had no living parents (*n* = 646) are not part of the sample. The sample was further restricted for the following reasons: first, 319 observations were eliminated because the respondent had no contact with either parent. Unfortunately, for these cases, no information on the distance between the child’s and the parent’s dwelling was available. Finally, some cases had to be eliminated due to item non-response on the variables included in the analytical models (*n* = 116) or missing migration distance due to unclarity regarding the place of residence mentioned by the respondent (*n* = 50). As this number is relatively small and imputations in this context are difficult or meaningless (for distance to parents or migration distance, for instance), these cases were dropped. Among these eliminated observations (in total *n* = 485; 3.30%), those with certain socio-demographic characteristics are over- or underrepresented. The excluded individuals were more likely to be members of the oldest birth cohort. Moreover, they received fewer years of education and were more likely to be born in Germany, even when conditioned on birth cohort. Moreover, the share of homeowners was larger in the analytical sample than the full dataset. The final analytical sample consisted of *N* = 14,215 observations for *n* = 5430 different individuals. A total of *n* = 2001 participants were selected for all four pairs of waves, while *n* = 840 were included in three, *n* = 1101 in two, and *n* = 1488 in one of the paired waves. The respondents were between 15 and 45 years old, with the highest frequencies concentrated around ages 22, 30, and 40.

To analyse the associations of the characteristics of the parent–child relationship with migration behaviour, we did not include the ties to both parents simultaneously (see Hank 2007, for instance). If an individual had contact with one parent only, or if only one of her/his parents was alive, this parent was selected (*n* = 3975). For an individual whose parents were not co-residing and who were living at different distances away from the child (*n* = 1193), only the bond to the parent who was living closer was analysed. We assumed that the parent who was living closer to the child contributed to the child’s social capital in that location. Obviously, when using this approach, other potential local ties might be ignored. However, to ensure that the model was simple enough that its results could be meaningfully interpreted, we decided to accept this drawback. For all other cases, we accounted for the parent–child characteristic that was found to be more pronounced, such as a higher frequency of instrumental support exchanges. This procedure was based on the assumption that—given that the child was living the same distance away from both parents—any tie to that location had an impact on the migration decision, regardless of to which of the parents the characteristic pertained.

### Dependent Variable

Data for the dependent variable describing internal migration behaviour were based on the dataset *biomob_ehc,* which is included in the scientific use file of *pairfam*. For each wave, the participant’s current place of residence, any changes of residence between the waves, the distance between each consecutive dwelling, and the start and end date of the residential episode in months were recorded (Brüderl et al. 2019b). The migration distance was calculated through a formula for orthodromes using coordinates of the two locations (see p. 74 in Brüderl et al. (2019b) for a more detailed explanation). Following previous research, internal migration was operationalised as a change of residence within Germany between a pair of consecutive waves *t* and *t* + 1 that equals or exceeds a distance of 40 km. To increase the number of observations, we used information on migrations recorded in wave *t* + 2 if a respondent participated in that wave but not in wave *t* + 1 (*n* = 510, which includes information of 95 observations taken from wave 10): if a change of residence was recorded to have happened within the interquartile range of the interview period of wave *t* + 1, we coded the respondent as having migrated between waves *t* and *t* + 1. In 19 cases, respondents reported to have migrated back and forth between two places of residence within the period from *t* to *t* + 1. These quick return migrations were not taken into consideration.

It should be noted that the data were left-censored: no information was available about migration histories. Most of those living far from their parents must already have migrated before, for example, upon leaving the parental home. (Some parents may also have migrated.) At the same time, some of those living close to their parents might have migrated towards them or returned to them after a previous move away, for example, in response to a support need. If that is the case, we might underestimate the importance of family for migration decisions.

### Independent Variables

Spatial proximity between the anchor and her/his parents was measured in travel time on a five-point scale ranging from *we live in the same house* (some respondents gave this answer even though they also reported not living in the same household) to *3* *h and longer*. We set the threshold of living geographically close to the parents to *less than 30* *min.*

For each of the mediating variables of intergenerational support exchange, one measure of the support given by the child to the parent and one measure for support received from the parent were included. Each measure (except the measure for care given to parents; see below) consisted of different items that were assessed on a five-point scale (*1: never, 2: seldom, 3: sometimes, 4: often, and 5: very often*), and we assumed that these items were related to the likelihood of migrating in an additive way. Therefore, we added up the relevant items of each measure and took their average for the sake of comparability between the different scales. The items were selected in accordance with previous research analysing intergenerational relationship types, which was based on the solidarity and conflict paradigm (e.g. Van Gaalen and Dykstra 2006).

The frequency of instrumental support was measured through the following three questions (displayed for giving support only; questions on receiving support were formulated analogously): *During the past 12* *months, how often did you give help in preparing documents such as tax forms or in taking care of official business? During the past 12* *months, how often did you give help to the following persons with shopping, housework, or yardwork? During the past 12* *months, how often did you give help to the following persons for the purpose of nursing or taking care of family members?* Additionally, our measure of giving instrumental support also included information about care given to the parent. To maintain consistency with the other instrumental support items, we coded this information in the following way: Cases in which the respondent reported that *the parent did not (need to) receive any help* were coded as *1* (*never*); cases in which the *respondent cared for the parent together with at least one other person*, such as a professional caregiver, friend, or neighbour, were coded as *3* (*sometimes*); and cases in which *the respondent was the sole caregiver* were coded as *5* (*very often*). The assignment of the values reflects our assumption that the burden of sole caregivers is usually larger compared to individuals who are able to share their care responsibilities. Both instrumental support variables were skewed to the right (1.17 for given; 1.51 for received).

The measure for emotional support exchange consisted of the following items: *During the past 12* *months, how often did you give advice regarding personal problems? During the past 12* *months, how often did you talk to the following persons about their worries and troubles?* Both variables were distributed almost normally, although slightly more emotional support was reported to be given than received. The frequency of receiving help with childcare from either parent was measured using a categorical variable distinguishing between individuals who *do not share a household with at least one child under age 15*, who have children under age 15 living with them and the *parent provides care never or rarely*, and who have children under age 15 living with them and the *parent provides care at least sometimes*. Even if the parent does not regularly care for her/his grandchildren, many families will still value frequent contact for the sake of maintaining a close relationship between grandchildren and grandparent (Oppelaar and Dykstra 2004).

We also included control variables indicating local capital and other factors that are known to influence migration. It is generally assumed that parents and children need to live close to each other in order to have frequent face-to-face contact (Larsen and Urry 2006), and that the frequency of such face-to-face contact partially explains the likelihood of migrating (Ermisch and Mulder 2018). Frequency of contact was operationalised as a dummy (*contact with parent at least several times per week*). This variable covered contact through visits, letters, phone calls, and similar forms of communication and was assumed to be a suitable overall measure for the intensity of parent–child contact. Unfortunately, the data did not allow us to distinguish between face-to-face and other forms of contact. Furthermore, living with a partner has been shown to deter migration (e.g. Cooke 2008). Conversely, having a living-apart-together (LAT) relationship in which the couple does not co-reside might enhance migration, because the individuals in the relationship might be more likely to migrate with the aim of moving in together (e.g. Krapf 2017). Partnership status is therefore operationalised through a variable with the categories *single, LAT,* and *lives with partner*. Having co-residing children is also known to deter individuals from migrating (e.g. Fischer and Malmberg 2001). In the model without mediators, we therefore control for sharing a household with children under age 15. Migration rates are also known to be strongly age-dependent (e.g. Bernard et al. 2014). As age is very unevenly distributed in pairfam due to its cohort structure, no measure for age but both *birth cohort* (*1971*–*1973* or *1981*–*1983* vs *1991*–*1993*) and *wave* were included. Taken together, these measures indicate the age of the respondent (e.g. Rabe-Hesketh and Skrondal 2012). Additionally, we controlled for *gender* and the educational level of the respondent measured in *years of education*. Generally, individuals with higher education tend to migrate more frequently than those with less education (Fischer and Malmberg 2001). Moreover, it has been shown that the spatial distance between parents and their adult children was strongly dependent on the degree of urbanisation of the area where they were living (Van der Pers and Mulder 2013), and that the intensity of family ties with regard to migration decisions varied between individuals with different cultural backgrounds (Zorlu 2009). We therefore included measures of whether the adult child was living in a rural area (*more than 20,000 inhabitants*) and was *born in Germany.* Finally, homeowners are known to be less likely to migrate (Fischer and Malmberg 2001). We therefore included a dummy variable *homeowner* which was coded 1 if the respondent reported owning or co-owning the home they were living in.

### Analytical Approach

**Fig. 1:**
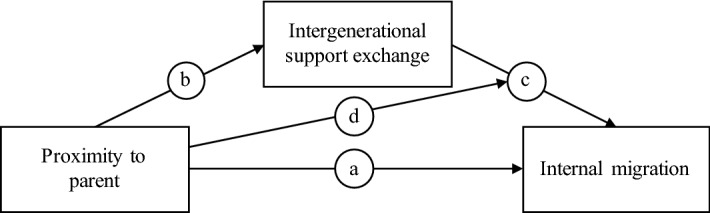
Conceptual model depicting the connections between dependent, independent, and mediating variables. *Source:* Own figure based on model of basic causal chain of mediation by Baron and Kenny (1986, Fig. 3) and the conditional process model by Hayes (2013, Fig. 12.1)

For our analyses, several logistic regression models were estimated following the classic approach of mediation analysis proposed by Baron and Kenny (1986).^1^ First, we tested hypothesis H1, which pertains to the main association between proximity to parents and the likelihood of migration. We ran a model with migration of at least 40 km as the dependent variable and spatial proximity to the parent as the main independent variable, while accounting for the control variables (Model 1) (arrow (a) in Fig. 1). Second, we explained each of the intergenerational support variables through spatial proximity using logistic regressions in order to test for their role as mediators (arrow (b) Models 2). Last, we sought to explain migration through the main independent variable (arrow (a)), the controls, and the mediators (arrow (c) (Model 3)). If the hypothesised mediator variables mediate the association of proximity to the parents and the likelihood of migration (H2, H3, and H4), the estimator for proximity should decrease in size and significance compared to the first model, while spatial proximity significantly predicts intergenerational support exchange (Baron and Kenny 1986). We calculated average marginal effects (AMEs) in order to be able to compare the sizes of the estimators between the different models (Mood 2010).

We assume that the association between intergenerational support exchange and the likelihood of migration differs by whether or not the parents live close to or far from the adult child. Conceptually, this can be understood as a moderated mediation with the main independent variable simultaneously being the moderator of the mediation (arrow (d) in Fig. 1). Statistically, an interaction between the moderator—distance to the parents—and the mediators can account for this (see Hayes 2013). Because migration is a rare event, we cannot estimate models including interaction terms for all of the mediators simultaneously due to a high risk of empty cells. Therefore, we estimated one full model without interaction terms and then ran several models including one moderator–mediator interaction at a time. For nonlinear models, interaction terms depend on the level not only of the moderation variable but also on the level of all the other covariates in the model. Therefore, it is not recommendable to only report one measure of the interaction (Wooldridge 2015). Instead, predicted probabilities of the likelihood of migration were calculated and plotted in graphs for relevant values of the support variables simultaneously accounting for the different slopes by proximity to the parent. The covariates for these graphs were set to their observed values for each individual. Because the observations of some individuals are used several times, we estimated standard errors that are robust to the clustering of observations over respondents.

Next to the main analysis, we ran several models as robustness checks and additional analyses. We report on the results of these models in a separate subsection (4.4).

## Results

### Descriptive Results

Table 1 displays the distribution of the dependent and independent variables, as well as the proportion of moves of at least 40 km, by their respective values. These descriptive results support our expectations. Overall, in 1.88% of all of the cases, a migration of at least 40 km occurred between two consecutive waves. The proportion of cases in which individuals migrated was much higher for those who did not live within less than 30 min travel time from their parents than for those who did. Additionally, the annual migration rate was slightly lower when the grandparents were taking care of their grandchildren at least sometimes compared to those helping only rarely or never, or if the respondents were not living with children under age 15.

**Table 1:** Descriptive statistics and proportion of migrations per category (*N* = 14,215)

	Mean		Proportion migrated ≥ 40 km	
Migrated ≥ 40 km	0.019			
Parent lives within < 30 min travel time: no	0.415		.035	
Yes	0.585		.007	
Instrumental support given (1–5)	1.718	(0.5675)		
Instrumental support received (1–5)	1.467	(0.6021)		
No children < 15 years in household	0.472		.033	
Parent provided childcare never or seldom	0.227		.007	
Parent provided childcare at least sometimes	0.302		.006	
Emotional support given (1–5)	2.784	(0.9149)		
Emotional support received (1–5)	2.706	(0.9627)		
Contact at least several times per week: no	0.369		.020	
Yes	0.631		.018	
Single	0.200		.029	
LAT	0.109		.057	
Lives with partner	0.691		.010	
Cohort 1991–1993	0.140		.069	
Cohort 1981–1983	0.388		.017	
Cohort 1971–1973	0.472		.005	
Wave 2	0.268		.012	
Wave 4	0.254		.018	
Wave 6	0.246		.020	
Wave 8	0.232		.026	
Female	0.569		.021	
Male	0.431		.017	
Years of education	13.338	(3.0112)		
Born in Germany: no	0.111		.008	
Yes	0.889		.020	
Lives in rural area (< 20.000 inh.): no	0.542		.021	
Yes	0.459		.016	
Homeowner: no	0.715		.026	
Yes	0.285		.001	

Standard deviations in parentheses

### Spatial Proximity to the Parent and the Likelihood of Migration

First, hypothesis H1 was tested using a logistic regression model without the mediator variables (Appendix 1, Model 1). The results support the hypothesis of a negative association between living close to a parent and the likelihood of migration. The AME of living within less than 30 min travel time from a parent on migrating was − 0.0200 (*p* = .000). Although this estimator seems to be tiny, it should be seen in relation to the small overall percentage of respondents who migrated (1.88%). Individuals who were living close to their parents thus are considerably less likely to migrate than individuals who were living farther away from their parents. Belonging to the older birth cohorts, sharing a household with children under age 15, and (partly) owning a home were also found to greatly reduce the propensity to migrate at least 40 km. By contrast, being in an LAT relationship compared to not having a partner, having a higher level of education, and being born in Germany were shown to increase the likelihood of migration significantly. These associations are in line with our expectations. The frequency of any kind of contact, gender, and living in a rural area were not, however, found to be significantly associated with migration behaviour.

### Support Exchange as Mediators

Second, the mediator variables were explained through proximity to the parents. As we would expect, we found that the frequency of instrumental support was significantly and positively related to living in close spatial proximity to the parent (Appendix 2). The results also indicated that individuals who were living within less than 30 min travel time from their parents and shared a household with children under age 15 were more likely to receive grandparental childcare at least sometimes and were less likely to receive this help never or seldom, than those living farther away. Giving emotional support was not significantly related to proximity to the parents. Surprisingly, individuals who lived close to their parents received less emotional support than those living far away. Hence, living in close proximity to a parent seems to be a precondition only for a more frequent exchange of instrumental support and receiving help with care for grandchildren.

**Fig. 2:**
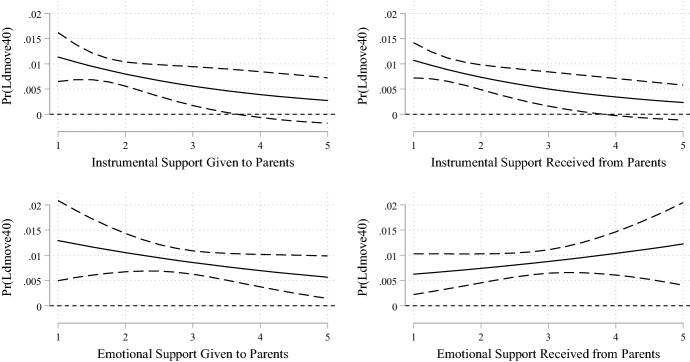
Predicted probabilities of migrating at least 40 km by frequency of intergenerational support exchange for those living close to the parents

**Fig. 3:**
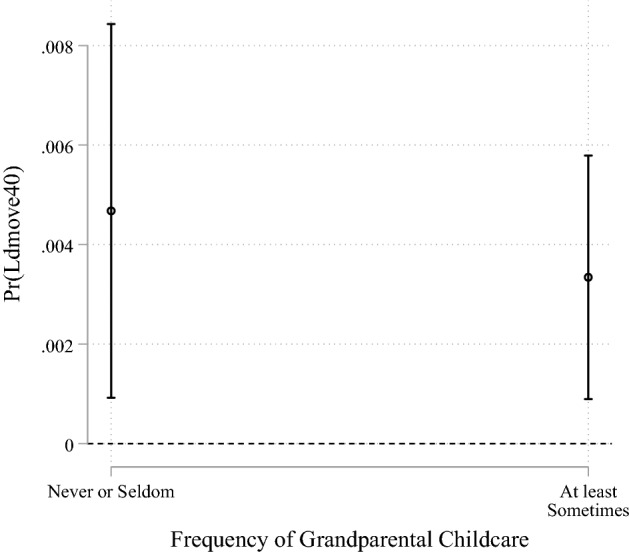
Predicted probabilities of migrating at least 40 km by frequency of receiving grandparental childcare for those living close to the parents and with co-residing children under age 15

The results for individuals who lived close to their parents that were derived from the models with interaction terms are shown in Fig. 2. The estimates of the control variables were not substantially affected by including the mediators, so we do not report them again. In line with hypothesis H2, the more instrumental support the individuals exchanged with their parents, the less likely they were to migrate when they lived close to them. This was also true of emotional support given to parents. Surprisingly, the more emotional support the individuals living close to their parents received from them, the higher was their migration propensity. The slopes of the associations of instrumental support exchange with migration likelihood appear to be slightly steeper compared to those for giving emotional support, indicating a more pronounced mediation effect of instrumental support. Whether or not individuals received care for their children from their parents at least sometimes compared to never or seldom did not seem to affect the likelihood of migration significantly for those who lived close to their parents and shared a household with children under age 15 (Fig. 3).

Including (non-interacted) measures for instrumental and emotional support exchange reduced the size of the estimator for geographic proximity to the parents on migration probability (ΔAME = − 0.0007 for the model without interaction terms). Thus, part of the main association seems to be explained through support exchanges between the generations. Because spatial proximity to the parent does not appear to be a precondition for giving emotional support, only giving and receiving instrumental support can be considered to mediate the association between living close and migrating in the way we expected it (support for H2). Moreover, receiving emotional support can also be viewed as a mediator, but the estimates point to the opposite of what we hypothesised. Finally, the inclusion of the mediators did not have a substantial impact on the significance or on the direction of the other variables of the model, even though it slightly changed the sizes of their coefficients. Concluding, giving emotional support was not significantly predicted through proximity to the parents, while receiving emotional support was not associated with a decrease but an increase in migration likelihood (no support for H3). Moreover, a higher frequency of grandparental childcare did not significantly predict a decreasing migration propensity (no support for H4).

### Robustness Checks and Additional Analyses

Arguably, the thresholds used for operationalising “moving away” and “living close to the parents” are to some degree arbitrary, and therefore disputable (Niedomysl et al. 2017). Although internal migration has often been defined using thresholds as high as 40 or 50 km (Mulder and Malmberg 2014; Ermisch and Mulder 2018, for instance), we ran our models using several combinations of different thresholds of moving distances (10, 20, and 30 km) and proximity to parents (less than 10 and less than 30 min travel time) to test for the robustness of the findings (not shown, available on request). Overall, the pattern of the results remained stable over the models. Including the mediator variables always decreased the strength and the significance of the negative AME of living close to one’s parents on the likelihood of migrating. In all models, the direction of the estimates of the mediators was consistent, despite some changes in the strengths and size of the confidence intervals. For a distance of 10 min travel time to the parent, receiving emotional support was not significantly predicted while giving it was. This is opposite to a distance of 30 min. Moreover, running models including all mediator–moderator interactions simultaneously with and without significant controls produced similar predicted probabilities of migrating for the values of the mediators compared to the models with one interaction at a time (despite obtaining slightly larger confidence intervals).

We additionally ran models using an operationalisation of migration that accounted for whether a change of residence between two waves was accompanied by a substantive increase in travel time to the parents (from less than 30 min to at least 1 h; results not shown, but available on request). The results were largely similar to the main models. However, except for receiving instrumental support, the associations between support exchange and migration likelihood were weaker and less statistically significant. It should be noted that the estimations for this analysis were less precise owing to a smaller number of migrations away from the parents than in our main analysis.

We also conducted additional analyses to account for a potential East–West divide within Germany (not shown). Despite the structural and attitudinal differences regarding formal childcare usage between East and West Germany (Schober and Spiess 2015), we did not find any significant East–West differences in the probability of migration, or significant interactions with grandparental help with childcare or living in a rural area. Furthermore, we ran a model in which we included the number of moves up to the 18th birthday, which was recorded in Wave 2. In previous research, the number of moves during childhood and adolescence was a strong predictor of the likelihood of moving for adults in Germany (Bernard and Vidal 2020). This number could be interpreted as an indicator of overall migration propensity or place attachment (Fischer and Malmberg 2001; Ermisch and Mulder 2018, for instance). However, the parameter for this indicator was not significant. Finally, an adult child’s emotional bond to her/his parent might influence the strength of the family tie, regardless of the frequency of emotional support exchanges. Including a measure for emotional closeness to the parent did, however, not change the results, and this measure was not found to be significantly associated with the likelihood of migration.

Furthermore, we investigated to what extent panel attrition might have biased our sample. Migration itself complicates re-locating and re-contacting the respondent at subsequent waves (Buck 2000; Lepkowski and Couper 2002). The attrition rates due to a wrong address or to a move of the sample person between two consecutive waves of the full pairfam sample decreased continuously from 1.9% (between Waves 1 and 2) to 0.5% (between Waves 5 and 6) (Suckow et al. 2010, 2011; Wich et al. 2012; Brix et al. 2013, 2014). In our analyses, a total of 10.95% of the baseline sample in Wave 2 could not be included due to panel attrition thereafter. A multinomial logistic regression of the competing risks of dropping out and of migrating at least 40 km between the four pairs of consecutive waves (see Appendix 3) revealed that the main associations between living close to the parent and support exchange were not affected by panel attrition. However, of the control variables significantly related to migration, years of education, being born in Germany, and homeownership were associated with the risk of dropping out. These findings are mostly in line with those of Müller and Castiglioni (2015) in their analysis of panel attrition in pairfam data.^2^

## Conclusion and Discussion

Despite the broad acknowledgement of the importance of the location of family members in migration decisions, the underlying mechanisms of why living in close proximity to family members tends to deter migration have only rarely been explored in previous research. In line with the existing literature, this study found support for the claim that there is a strong link between adult children’s local ties to their parents and their likelihood of moving away in the geographic context of Germany. Living within less than 30 min travel time from one’s parent was shown to strongly reduce the likelihood of migrating at least 40 km. Additionally, we were able to partly explain this association mainly through instrumental support flows using mediation analysis within a cost–benefit framework of migration theory.

We anticipated, first, that any type of support would be exchanged more frequently—in both directions—if adult children lived close to their parents. Second, we expected that any support exchange would be negatively associated with the likelihood of migrating among those living close to their parents. However, opposite to our expectations, the frequency of children giving emotional support to the parents did not depend on the distance between their residential locations, and those who lived close to their parents received emotional support *less* frequently than those who lived further away. Other than for instrumental support, spatial proximity might not be a precondition for exchanging emotional help. Keeping in touch via telephone, smartphone, or the Internet might indeed be sufficient for maintaining the relationship. Recent findings of Steinbach et al. (2019) are in line with this idea: on an aggregate level, they found a high stability regarding frequency of contact and emotional closeness within intergenerational relationships in Germany between 1996 and 2014 despite an increasing spatial distance between older parents and their children. Also opposite to what we expected, an increasing frequency of receiving emotional support was associated with *higher* propensities of migrating. A speculative explanation of this finding could be that adult children who receive emotional support on a regular basis might be more confident to successfully adapt to a new location and might, therefore, be more likely to actually migrate. Given that many German grandparents feel the obligation to help with childcare (Hank and Buber 2009) and in fact provide such help (Mahne and Klaus 2017), it is surprising that we also did not find a significant association between grandparental caregiving and the likelihood of migrating. It is possible that grandparental childcare is only important at preschool ages, but our data did not allow us to distinguish between preschool and school-aged children due to small numbers. Moreover, face-to-face contact with the grandparents might matter next to effective help with childcare.

It should be acknowledged that reversed causality may be an issue in our findings. For example, families or individuals might move closer to their parents in anticipation of or in response to becoming parents as they know they cannot live too far away from their parents if they want to receive informal help with childcare (Michielin et al. 2008). Although we were not able to address this issue empirically within the scope of this paper, we again emphasise the theoretical assumption that spatial proximity to the parents is a precondition for exchanging instrumental support. While support can only be given if spatial proximity has already been established, it is likely to function as a deterrent to migration thereafter. Ermisch and Mulder (2018), along with Heylen et al. (2012), found empirical indications of this direction of causality in their analyses.

There are some limitations concerning our data, sample, and variable selection. The cohort structure of the data results in an awkward age distribution, and the analytical sample was not completely representative regarding essential socio-demographic variables, such as education or homeownership. Furthermore, due to panel attrition, respondents who were highly educated, female, born in Germany, or a homeowner were overrepresented in our sample. The number of migrants in this sample is also rather small. This easily overstretches the data, and the number of variables and combinations of variables that could be included in the models was limited. Moreover, other relevant local ties, such as ties to siblings and friends (Belot and Ermisch 2009; Mulder and Van der Meer 2009) or to work (Fischer and Malmberg 2001), could not be accounted for due to a lack of suitable variables or proxies. Parents are only one potential source of social capital. Thus, other local ties might compete with the tie to a parent, or they might further strengthen an individual’s bond to her/his current place of residence (Mulder 2018). Therefore, we cannot be completely sure if the associations that we measured are related to ties to the parents or to some other source of local capital.

Nonetheless, our findings underline the importance of family as a manifestation of local social capital in general if we consider that strong ties between parents and children are likely to indicate strong ties to other family members, such as siblings (see Hank and Steinbach 2018; de Bel et al. 2019). Moreover, because the analysis focusses on the binding effect of parents and does not examine parents as an attraction factor for migration, we potentially underestimate the overall importance of parents as a factor in migration decisions.

In conclusion, we were able to extend existing research on the relevance of family ties for internal migration decisions by including direct measures of intergenerational support exchanges between parents and their adult children using longitudinal survey data. Although a large part of this association has yet to be explained, the results highlight the importance of the social environment in internal migration decisions. More importantly, this work contributes to our understanding of the mechanisms underlying the binding effect of family. We used several points in time in a migration theory-based mediation analysis to bring some light into the black box of why living close to family members, and to parents in particular, deters migration. Adult children seem to value the instrumental support they exchange with their parents when evaluating whether to move away. Future research might build on the presented results, especially with regard to the inclusion of further relationship characteristics and other local ties, as well as relationships with siblings, other family members, and other members of the social network. Conducting the analyses for other geographical contexts might reveal to what extent the welfare state regime interacts with the influence of instrumental support flows in migration decisions (e.g. Hank and Buber 2009). It would also be helpful to use data from an older sample, in order to be able to investigate the role of caring for parents in migration decisions. Merely acknowledging that having family ties deters migration is not enough if we want to fully grasp how migration decisions are formed—let alone how migration processes are shaping cities and regions.

## Appendix 1

See Table 2.

**Table 2:** Logistic regressions of migration with and without mediators (AMEs) (no X–M interactions) *Source*: pairfam, release 10.0 (Brüderl et al. 2019a)

	(1)	(2)
Parent lives within < 30 min travel time	− 0.0200***	− 0.0193***
	(0.0024)	(0.0025)
Instrumental support given	–	0.0032
		(0.0023)
Instrumental support received	–	− 0.0041
		(0.0022)
Care for grandchild received (ref. never or rarely)		
Parent provided childcare at least sometimes	–	0.0019
		(0.0043)
No children < 15 years in household	–	0.0066*
		(0.0034)
Emotional support given	–	− 0.0033*
		(0.0016)
Emotional support received	–	0.0036*
		(0.0016)
Contact with parent at least several times a week	− 0.0021	− 0.0029
	(0.0024)	(0.0027)
Partnership status (ref. single)		
LAT	0.0109**	0.0107**
	(0.0038)	(0.0038)
Living with partner	− 0.0046	− 0.0044
	(0.0026)	(0.0026)
Child < 15 years in household	− 0.0063*	–
	(0.0027)	
Cohort (ref. 1991–1993)		
1981–1983	− 0.0258***	− 0.0247***
	(0.0053)	(0.0054)
1971–1973	− 0.0336***	− 0.0322***
	(0.0051)	(0.0052)
Wave (ref. two)		
Wave 4	− 0.0002	0.0004
	(0.0039)	(0.0039)
Wave 6	− 0.0041	− 0.0038
	(0.0037)	(0.0037)
Wave 8	− 0.0022	− 0.0023
	(0.0039)	(0.0039)
Male	− 0.0031	− 0.0025
	(0.0023)	(0.0024)
Years of education	0.0015**	0.0015**
	(0.0005)	(0.0005)
Born in Germany	0.0088**	0.0087**
	(0.0032)	(0.0032)
Rural area	0.0042	0.0043
	(0.0025)	(0.0025)
Homeowner	− 0.0177***	− 0.0175***
	(0.0020)	(0.0020)
Migration imputed from t + 2	− 0.0113	− 0.0112
	(0.0070)	(0.0069)
Pseudo-R^2^	0.1778	0.1821
*N*	14,215	14,215
Number of clusters (*n*)	5430	5430

Standard errors in parentheses (corrected for clustered individuals); *** *p* < .001, ** *p* < .01, * *p* < .05

## Appendix 2

See Table 3.

**Table 3:** AMEs of living close to parents on mediator variables of bivariate linear regressions^a^ (refers to path (b) of the conceptual model depicted in Fig. 1). *Source*: pairfam, release 10.0 (Brüderl et al. 2019a)

Independent	Mediator variables (dependent)	AMEs
Parent lives within < 30 min travel time	Instrumental support given	0.1845***
		(0.0128)
	Instrumental support received	0.2304***
		(0.0125)
	Parent provided childcare at least sometimes (ref. never or rarely)^a^	0.3672***
		(0.0149)
	Emotional support given	0.0316
		(0.0212)
	Emotional support received	− 0.0836***
		(0.0233)

Standard errors in parentheses (corrected for clustered individuals); ****p* < .001; ***p* < .01; **p* < .05; *N* = 14,215 observations clustered over *n* = 5430 individuals; ^a^to predict grandparental childcare, we conducted a bivariate logistic regression for individuals with co-residing children < 15 years (*N* = 7510 with *n* = 2883 clustered individuals)

## Appendix 3

See Table 4.

**Table 4:** Relative risk ratios of migration versus panel attrition. *Source*: pairfam, release 10.0 (Brüderl et al. 2019a)

	Migration versus panel attrition (ref. not migrated)	
	Drop-out	Migrated ≥ 40 km
Parent lives within < 30 min travel time	0.9997	0.2981***
	(0.0600)	(0.0492)
Instrumental support given	1.0074	1.2149
	(0.0562)	(0.1570)
Instrumental support received	0.9499	0.7775*
	(0.0477)	(0.0961)
Care for grandchild received (ref. never or rarely)		
Parent provided childcare at least sometimes	1.0001	1.1842
	(0.0774)	(0.3730)
No children < 15 years in household	0.9791	1.5295
	(0.0753)	(0.3706)
Emotional support given	0.9576	0.8190*
	(0.0346)	(0.0749)
Emotional support received	1.0374	1.2369*
	(0.0358)	(0.1122)
Contact with parent at least several times a week	0.9699	0.8561
	(0.0609)	(0.1260)
Partnership status (ref. single)		
LAT	0.9663	1.6595**
	(0.0943)	(0.2748)
Living with partner	0.9617	0.7666
	(0.0693)	(0.1256)
Cohort (ref. 1991–1993)		
1981–1983	0.9525	0.3544***
	(0.0900)	(0.0649)
1971–1973	0.8672	0.1802***
	(0.0897)	(0.0413)
Wave (ref. two)		
Wave 4	0.6256***	1.0370
	(0.0411)	(0.2094)
Wave 6	0.5142***	0.8091
	(0.0369)	(0.1693)
Wave 8	0.3635***	0.8792
	(0.0301)	(0.1895)
Male	1.1325*	0.8650
	(0.0629)	(0.1193)
Years of education	0.9464***	1.0881**
	(0.0088)	(0.0311)
Born in Germany	0.7527***	1.8590*
	(0.0581)	(0.5437)
Rural area	1.0080	1.2834
	(0.0541)	(0.1720)
Homeowner	0.8598*	0.1475***
	(0.0567)	(0.0696)
*N*	15,963	15,963
Number of clusters(*n*)	6366	6366

Standard errors in parentheses (corrected for clustered individuals); *** *p* < .001, ** *p* < .01, * *p* < .05

Pseudo-*R*^2^ = 0.0579

## Footnotes

1 Structural equation models produce the same output. However, for the sake of simplicity, presentation of the results, and computing time we employed separate logistic regressions.

2 Unlike us, Müller and Castiglioni (2015) also found significantly greater attrition among those in living-apart-together relationships than among singles. This difference may be related to the fact that these authors examined all individuals of the three pairfam cohorts, rather than just those not co-residing with their parents.
